# Knockdown of *GmVQ58* encoding a VQ motif-containing protein enhances soybean resistance to the common cutworm (*Spodoptera litura* Fabricius)

**DOI:** 10.1093/jxb/eraa095

**Published:** 2020-02-20

**Authors:** Xiao Li, Rui Qin, Qing Du, Linyan Cai, Dezhou Hu, Haiping Du, Hui Yang, Jiao Wang, Fang Huang, Hui Wang, Deyue Yu

**Affiliations:** 1 National Center for Soybean Improvement, National Key Laboratory of Crop Genetics and Germplasm Enhancement, Jiangsu Collaborative Innovation Center for Modern Crop Production, Nanjing Agricultural University, Nanjing, China; 2 School of Life Sciences, Guangzhou University, Guangzhou, China; 3 The James Hutton Institute, UK

**Keywords:** Common cutworm, GmVQ58, GmWRKY32, resistance, soybean, *VQ* gene

## Abstract

Plants have evolved complex defense mechanisms to withstand insect attack. Identification of plant endogenous insect resistance genes is of great significance for understanding plant–herbivore interactions and improving crop insect resistance. Soybean (*Glycine max* (L.) Merr.) is an important crop that is often attacked by the common cutworm (CCW) (*Spodoptera litura* Fabricius). In this study, based on our transcriptomic data, the gene *GmVQ58*, encoding a FxxxVQxxTG (VQ) motif-containing protein, was cloned and characterized. This gene showed the highest expression in the leaves and roots and was up-regulated significantly after CCW attack. Constitutive expression of *GmVQ58* rescued the susceptibility of an Arabidopsis mutant to CCW, and interference of *GmVQ58* in soybean hairy roots enhanced the resistance to CCW. Furthermore, GmVQ58 was localized to the nucleus and physically interacted with the transcription factor GmWRKY32. The expression of two defense-related genes, *GmN:IFR* and *GmVSPβ*, was up-regulated in *GmVQ58-*RNAi lines. Additionally, the promoter region of *GmVQ58* was likely selected during domestication, resulting in different expression patterns in cultivated soybeans relative to wild soybeans. These results suggest that silencing *GmVQ58* confers soybean resistance to CCW.

## Teaser

The soybean gene *GmVQ58* responds to common cutworm infestation and its protein interacts with the transcription factor GmWRKY32. Silencing *GmVQ58* enhances soybean defense against common cutworm by up-regulating expression of two resistance genes.

## Introduction

Plants are constantly exposed to numerous abiotic and biotic stress factors during their life cycle, of which insect attack is a severe stress that reduces plant growth and development and agricultural output. Soybean (*Glycine max* (L.) Merr.) is one of the most important crops, providing humans with high-quality protein and abundant oils. This plant faces threats from various herbivorous insects of which the common cutworm (CCW) (*Spodoptera litura* Fabricius) is one of the major pests in southern China and southwestern Japan, feeding on soybean leaves, flowers, pods, and young stems (Cui *et al.*, 1997; Komatsu *et al.*, 2004). Under millions of years of selection pressure generated by insects, plants have evolved complex defense systems to protect themselves from attack (Kessler and Baldwin, 2002; Erb and Reymond, 2019). Understanding the mechanism of insect resistance is important for breeding insect-resistant plants.

The rapid development of next-generation genome sequencing technologies, such as RNA sequencing (RNA-seq), has provided effective tools to explore the mechanisms of gene regulatory networks and identify relevant genes. For example, in rice a transcription factor was identified that coordinates internode elongation and photoperiodic signals, by analysing the transcriptome profiles of two varieties with different photoperiod sensitivities under short-day treatment (Gómez-Ariza *et al.*, 2019). In tomato transcriptome analysis revealed potential mechanisms for inhibition of intumescence by ultraviolet radiation (Wu *et al.*, 2017). In soybean, our group previously initiated a study on the defense responses of cultivated soybeans to CCW via RNA-seq (Wang *et al.*, 2015*d*). Through database analysis, *GmVSPβ* (encoding a vegetative storage protein), *GmN:IFR* (encoding an NADPH:isoflavone reductase) and *GmWRKY32* (encoding a WRKY-type transcription factor) showed differential expression patterns between CCW-resistant and CCW-susceptible soybean lines. *GmVSPβ* and *GmN:IFR* positively regulate plant resistance to CCW in tobacco (Wang *et al.*, 2015*d*), and GmWRKY32 strongly activated the transcription of these genes in Arabidopsis protoplasts (Wang *et al.*, 2015*c*). In addition, many other differentially expressed genes were detected in our transcriptome database; these genes were involved in several important cellular processes, including primary signaling related to defense, transcriptional regulation, and secondary metabolism. Among these genes, a *VQ* gene showed markedly higher expression levels in a CCW-susceptible soybean accession than in a CCW-resistant soybean accession.

*VQ* genes are known for their proteins’ containing a conserved FxxxVQxxTG motif (VQ motif) (Cheng *et al.*, 2012). These genes are plant-specific and form multigene families with 34, 39, 18, and 74 members in Arabidopsis, rice, grape, and soybean, respectively (Cheng *et al.*, 2012; Kim *et al.*, 2013; Wang *et al.*, 2014; Wang *et al.*, 2015*b*). Most of these genes are heterogeneously distributed on chromosomes and do not contain introns. To date, functional analysis of *VQ* members has mainly focused on the model plant Arabidopsis. Arabidopsis *AtVQ21*, encoding one substrate of mitogen-activated protein kinase 4, functions as a positive regulator in plant defense against *Pseudomonas syringae* (Petersen *et al.*, 2010). *AtVQ12* is highly responsive to *Botrytis cinerea* infection and has functional redundancy with *AtVQ29* in the control of plant basal resistance against *B*. *cinerea* (Wang *et al.*, 2015*a*). *AtVQ23* and *AtVQ16*, encoding two sigma factor binding proteins, were also reported to regulate plant defense against *B*. *cinerea* in a redundant manner (Lai *et al.*, 2011). Loss-of-function of *AtVQ22* (*JAV1*) leads to increased plant resistance against a variety of insects and pathogens, such as *Spodoptera exigua*, *Bradysia impatiens*, aphids, and *B*. *cinerea* (Hu *et al.*, 2013*a*). In soybean, two *VQ* genes, *GmVQ35* and *GmVQ47*, were reported to participate in plant resistance against *B. cinerea* (Zhou *et al.*, 2016). In addition to responding to biotic stresses, *VQ* genes play an important role in plant responses to abiotic stress. For instance, in Arabidopsis, overexpression of *AtVQ9* causes high susceptibility of transgenic Arabidopsis to salt stress (Hu *et al.*, 2013*b*). *AtVQ15*, which encodes a calmodulin-binding protein, is necessary for osmotic stress tolerance in plant seedlings (Perruc *et al.*, 2004). In banana fruit, *MaVQ5* was reported to be cold-responsive and may function as a repressor to antagonize *MaWRKY26* in relation to the methyl jasmonate-mediated cold stress response (Ye *et al.*, 2016). Moreover, some Arabidopsis *VQ* genes were reported to regulate plant growth and development. *AtVQ14* is strongly expressed in early endosperm development, and only small seeds are produced in *vq14* mutants, suggesting that *AtVQ14* not only regulates endosperm development but also affects seed size (Wang *et al.*, 2010). Loss-of-function of *AtVQ8* leads to pale-green and stunted-growth phenotypes, and the overexpression of *AtVQ17* and *AtVQ18* suppresses plant growth and development (Cheng *et al.*, 2012).

Although *VQ* genes have been well studied in Arabidopsis, few studies have reported the roles of soybean *VQ* genes, especially their function in insect resistance. In this study, we isolated and characterized a soybean *VQ* gene, *GmVQ58.* We analysed the genomic sequence of the gene and characterized its protein localization. The expression pattern of this gene in various tissues and leaves after CCW feeding was also investigated, and its protein interaction with GmWRKY32 was demonstrated *in vivo*. Two force-feeding trials were carried out to evaluate the resistance of *GmVQ58* transgenic Arabidopsis and soybean hairy roots. Additionally, the promoter region of *GmVQ58* was analysed for sequence diversity in wild soybeans, landraces and improved cultivars. Our work provides insight into the role and mechanism of *GmVQ58* in plant defense against CCW, which may lay the foundations for breeding soybean for insect resistance.

## Materials and methods

### Plant materials and common cutworm induction treatments

The soybean cultivar Williams 82 was grown in a growth room with a 16 h–8 h day–night photoperiod, a daytime temperature of 25 °C and a night-time temperature of 24 °C. To identify the expression level of *GmVQ58* in various tissues, leaves, stems, roots, flowers, pod shells, and seeds of soybean were sampled at different developmental stages: leaves, stems, and roots were sampled at the V4 stage; mature flowers were collected at the R2 stage; and pod shells and seeds were harvested 15 d after flowering. CCW induction treatments using soybean seedlings from the V4 stage were performed as reported (Fan *et al.*, 2012). Leaves from both control and treatment plants were collected at 1, 6, 12, 24, 48, and 72 h after CCW attack for isolation of total RNA.

The Arabidopsis ecotype Columbia 0 (Col-0) plants and the *jav1* mutants (SALK_146039C) used in this study were obtained from the Arabidopsis Biological Resource Center (ABRC, Columbus, OH, USA). The T-DNA was inserted into the promoter of the *JAV1* gene, approximately 600 bp upstream of the ATG (translational start codon) (Ali *et al.*, 2019). Arabidopsis plants were grown in a growth room with a 14 h day and 10 h night photoperiod, daytime temperature of 23 °C and night-time temperature of 22 °C. CCW induction treatments in 21-day-old transgenic Arabidopsis were carried out using a previously described protocol (Liu, 2015). Leaves from transgenic Arabidopsis were attacked by CCW and left for 1 h before histochemical β-glucuronidase (GUS) staining. Leaves from un-attacked transgenic Arabidopsis were used as controls.

### Analysis of differentially expressed soybean *VQ* genes

Twenty-nine soybean *VQ* genes were present in our previously reported analysis of the transcriptome responses of two cultivated soybean lines to CCW attack (Wang *et al.*, 2015*d*). Four treatments were used in this experiment (treatment of the resistant soybean lines (RK) at 5 d and susceptible soybean lines (SK) at 1 d and their corresponding controls (RCK) and (SCK)). The sequence data were downloaded from the NCBI database (https://www.ncbi.nlm.nih.gov/sra/SRP049638). We filtered the raw reads, assembled the transcriptome, blasted the reference database, and quantified the gene expression (reads per kilobase per million mapped reads (RPKM)) as described by Wang *et al.* (2015*d*). Differentially expressed *GmVQs* were identified by a statistical comparison using the rigorous algorithm method (Audic and Claverie, 1997). The screening conditions were set as follows: false discovery rate ≤0.001 and an absolute value of log_2_ fold change between susceptible and resistant lines ≥1.5. A heat map based on gene expression was generated by the R software package ‘heatmap’ (https://cran.r-project.org/web/packages/heatmap3/index.html).

Another transcriptomic dataset for wild soybeans responding to CCW attack was also used in this study (Du *et al.*, 2019). The resistant lines were sampled at 1 d (RK-1d, RCK-1d) and 2 d (RK-2d, RCK-2d) after induction, while the susceptible lines were collected at 1 d (SK-1d, SCK-1d) and 3 d (SK-3d, SCK-3d) after induction. The sequence data were also downloaded from the NCBI database (https://www.ncbi.nlm.nih.gov/sra/PRJNA493962). The filtration of raw reads, alignment to the reference genome and quantification of gene expression were performed as reported by Du *et al.* (2019). Differential gene expression was determined using the DESeq R package (Anders and Huber, 2010). The screening standards for differentially expressed *VQ* genes in wild soybeans were consistent with those in cultivated soybeans.

The analysis of the two transcriptomes was conducted in cooperation with Beijing Genomics Institute (BGI), China.

### Cloning of the *GmVQ58* gene

The full-length cDNA of *GmVQ58* (*Glyma.14g002800*) was PCR-amplified from the leaf cDNA of soybean cultivar Williams 82 (primers shown in see Supplementary Table S1 at *JXB* online). The PCR products were gel-purified (Axygen, USA) and sequenced by Thermo Fisher Scientific (Shanghai, China).

### Sequence and phylogenetic analysis

For the names of the soybean and Arabidopsis *VQ* genes, refer to Wang *et al.* (2014) and Cheng *et al.* (2012); the protein sequences were obtained from Phytozome (https://phytozome.jgi.doe.gov/pz/portal.html). Putative *Physcomitrella patens* VQ protein sequences were obtained using the VQ motif as a keyword to search the *Physcomitrella patens* database in Phytozome. The sequence alignment was analysed using ClustalX software version 1.83 (Jeanmougin *et al.*, 1998). A neighbor-joining (NJ) phylogenetic tree was constructed based on protein sequences with MEGA 6.0 software using the following parameters: bootstrap (1000 replications), *p*-distance, uniform rates, and pairwise deletion (Tamura *et al.*, 2013). The best Arabidopsis hit for the GmVQ58 protein was determined by BLASTP analysis against the Arabidopsis proteins (http://www.arabidopsis.org/). GSDS (http://gsds.cbi.pku.edu.cn) was used to analyse the gene structure.

### Gene expression analysis

Total RNA was isolated from Arabidopsis and soybean using the RNAsimple Total RNA Kit (TianGen, Beijing, China), and first-strand cDNA was reverse-transcribed with the PrimeScript^TM^ 1st Strand cDNA Synthesis Kit (TaKaRa, Dalian, China). Quantitative real-time polymerase chain reaction (qRT-PCR) was used for gene expression analysis using an ABI 7500 system (Applied Biosystems, Carlsbad, CA, USA) with Aceq qPCR SYBR Green Master Mix (Vazyme Biotech Co., Nanjing, China), and qRT-PCR data were analysed using the $2_{}^{-\Delta \Delta C_{t}}$ method (Livak and Schmittgen, 2001). Three biological and three technical replicates were used in these experiments. The soybean *tubulin* gene (*Glyma.03g124400*) and Arabidopsis *tubulin* gene (*At5g62690*) were used as internal controls to normalize the samples. All specific primers used for the gene analysis are listed in Supplementary Tables S1, S2. Student’s two-tailed *t*-test was used for statistical analysis.

### Histochemical β-glucuronidase staining

Genomic DNA was extracted from leaves of the soybean cultivar Williams 82 with a DNAsecure Plant Kit (TianGen, Beijing, China). The 2184-bp region upstream of the ATG of *GmVQ58* was cloned from the leaf DNA using special primers (shown in Supplementary Table S1) and subsequently inserted into the pCAMBIA1381z vector. Then, the recombinant plasmid *GmVQ58*_*pro*_*:GUS* was introduced into Arabidopsis via the floral dip method (Clough and Bent, 1998). GUS staining in T_3_ transgenic plants was performed using a previously described protocol (Chao *et al*., 2014).

### Subcellular localization

The ORF of *GmVQ58* without a stop codon was cloned into the pFGC5941 vector with the green fluorescent protein (GFP) gene downstream of the CaMV 35S promoter. Then, the recombinant vector 35S:GmVQ58-GFP and control (empty vector 35S:GFP) were transformed into tobacco (*Nicotiana benthamiana*) leaves for transient expression. The GFP signal was monitored by confocal laser scanning microscopy (Leica TCS SP2, Mannheim, Germany).

### Construction of the *GmVQ58* overexpression vector for Arabidopsis transformation

To construct the pMDC83-*GmVQ58* overexpression vector, the coding sequence (CDS) of *GmVQ58* was cloned into the pMDC83 vector by Gateway^TM^ technology (Thermo Fisher Scientific, Shanghai). Then, the recombinant vector driven by the double CaMV 35S promoter was introduced into *Agrobacterium tumefaciens* strain EHA105 and subsequently transformed into Arabidopsis using the floral dip method (Clough and Bent, 1998). Since the pMDC83 vector contained the hygromycin B gene (HygB), the transgenic Arabidopsis plants were screened on Murashige and Skoog medium containing 40 μg ml^−1^ HygB. The HygB-resistant plants were then transplanted to soil for further molecular identification at both the genomic and the transcriptional level. Three homozygous T_3_ transgenic lines were chosen for phenotypic analysis and evaluation of resistance to CCW. The primers used in this experiment are listed in Supplementary Table S2.

### Overexpression and suppression of *GmVQ58* in soybean hairy roots

The pMDC83-*GmVQ58* overexpression vector was also used in this experiment. Considering that the length of the *GmVQ58* CDS was less than 500 bp, to meet the length requirement of interference fragments, the full CDS of *GmVQ58* was cloned into the vector pB7GWIWG2(II) to generate the pBI-*GmVQ58-*RNAi vector. Then, pMDC83-*GmVQ58*, pBI-*GmVQ58*-RNAi, and their separate empty vectors were transformed into *Agrobacterium rhizogenes* strain K599. The soybean cultivar ‘Jack’ was used as a hairy root transgenic acceptor for its high efficiency in transformation. Four different transformed *Agrobacterium rhizogenes* strains were separately inoculated into Jack cotyledons as described previously (Du *et al.*, 2016). The cotyledons were grown on white medium containing 500 μg ml^−1^ carbenicillin disodium and 50 μg ml^−1^ cefotaxime disodium at 25 °C in the dark. After 25 d, transgenic hairy roots were formed from the abdomen of inoculated cotyledons and confirmed by PCR and qRT-PCR analysis (primers shown in Supplementary Table S1).

### Force-feeding trial with common cutworm

Third-instar CCW larvae of a similar size were selected and starved for 12 h before participating in the force-feeding trial. In one feeding experiment, 15 mature rosette leaves from 28-day-old Arabidopsis plants for each line were placed in a plastic Petri dish. Five larvae were raised in one plastic Petri dish for 4 d. Another force-feeding trial lasted 6 d using soybean hairy roots. Five larvae were randomly placed in a plastic Petri dish and raised on the hairy roots from one soybean genotype.

Five CCW larvae from each dish (for each Arabidopsis line or soybean genotype) were weighed every day, except for the first day after feeding. The leaves or hairy roots in each Petri dish were replaced with fresh samples every day. Each Arabidopsis line or soybean genotype had three independent replicates. The relative growth rate (RGR) of CCW larvae were calculated using the formula RGR=Δ*W*/(*W*_0_×*T*), where Δ*W*, *W*_0_, and *T* represent change in insect weight, initial insect weight, and feeding period in days, respectively (Farrar *et al.*, 1989). Two-tailed *t*-tests were used for statistical analysis.

### Yeast two-hybrid assay

A yeast two-hybrid (Y2H) assay was performed using the Matchmaker™ Gold Yeast Two-Hybrid System (Clontech, USA). The CDS of *GmVQ58* was cloned into the vector pGBKT7 (BD) to produce the construct BD-GmVQ58. Additionally, the CDS of *GmWRKY32* (*Glyma.02g115200*) was PCR-amplified from the leaf cDNA of the soybean cultivar Williams 82 at 1 d after CCW attack and was subcloned into the vector pGADT7 to produce the construct AD-GmWRKY32 (primers shown in Supplementary Table S1). The transformed yeast cells were first grown on plates containing double-dropout media (−Leu/−Trp) and then transferred to higher-stringency plates containing quadruple-dropout media (−Leu/−Trp/−His/−Ade). Combinations of pGBKT7-53 (BD-53) and pGADT7-T (AD-T) were used as positive controls, while the negative controls were pGBKT7-lam (BD-lam) and AD-T. Plates used for the identification of protein interactions were supplemented with X-α-Gal to visualize the interactions.

### Bimolecular fluorescence complementation assay

The CDSs of *GmVQ58* and *GmWRKY32* were cloned into the vectors SPYNE173 and 35S-SPYCE (M) to produce the constructs GmVQ58-YFP^N^ and GmWRKY32-YFP^C^, respectively, using the primers shown in Supplementary Table S1. Then, the constructs GmVQ58-YFP^N^ and GmWRKY32-YFP^C^ were co-transformed into tobacco (*N. benthamiana*) leaves for transient expression. The yellow fluorescent protein (YFP) signal was monitored by confocal laser scanning microscopy (Leica TCS SP2, Mannheim, Germany).

### Promoter diversity analysis

Promoter diversity analysis was conducted by using previously published whole-genome sequencing data of 302 soybean accessions, which included 62 wild soybeans, 130 landraces and 110 improved cultivars (Zhou *et al.*, 2015). The single-nucleotide polymorphism (SNP) data were downloaded online (https://figshare.com/articles/Soybean_resequencing_project/1176133) and then imported into the program VCFtools (Danecek *et al.*, 2011). We used the gene name of *GmVQ58* as a keyword to search Phytozome (https://phytozome.jgi.doe.gov/pz/portal.html) and obtained the gene’s physical location in the soybean reference genome (*Glycine max Wm82.a1.v1*) (Schmutz *et al.*, 2010). According to the physical location, the SNP information for the 2184-bp region upstream of the ATG of *GmVQ58* was obtained by VCFtools. In VCFtools, the nucleotide diversity (π) and Tajima’s *D* were calculated on the basis of the SNP information within the *GmVQ58* promoter between accessions in each subpopulation; π represents the average number of nucleotide differences per site between two sequences (Choudhury *et al.*, 2014). In addition, Tajima’s *D* test was used to evaluate the neutrality of the polymorphisms (Tajima, 1989). The visualization of aligned SNPs within the *GmVQ58* promoter in 302 soybean accessions was conducted by GeneDoc software (Aguilar-Martínez *et al.*, 2007).

The raw sequencing data containing the 2184-bp promoter region of *GmVQ58* in 302 accessions (https://www.ncbi.nlm.nih.gov/sra/?term=SRP045129) were mapped to the reference genome as reported by Zhou *et al.* (2015) and then converted to BAM format via SAMtools software (Li *et al.*, 2009). The BAM files were used to acquire the 2184-bp region sequence in each accession by IGVtools software (Robinson *et al.*, 2011). The relative conservation value of the *GmVQ58* promoter sequences among the accessions was calculated by DnaSP 6 software (Rozas *et al.*, 2017).

## Results

### Identification and phylogenetic tree analysis of differentially expressed *GmVQs* in the RNA-seq database

**Fig. 1.**
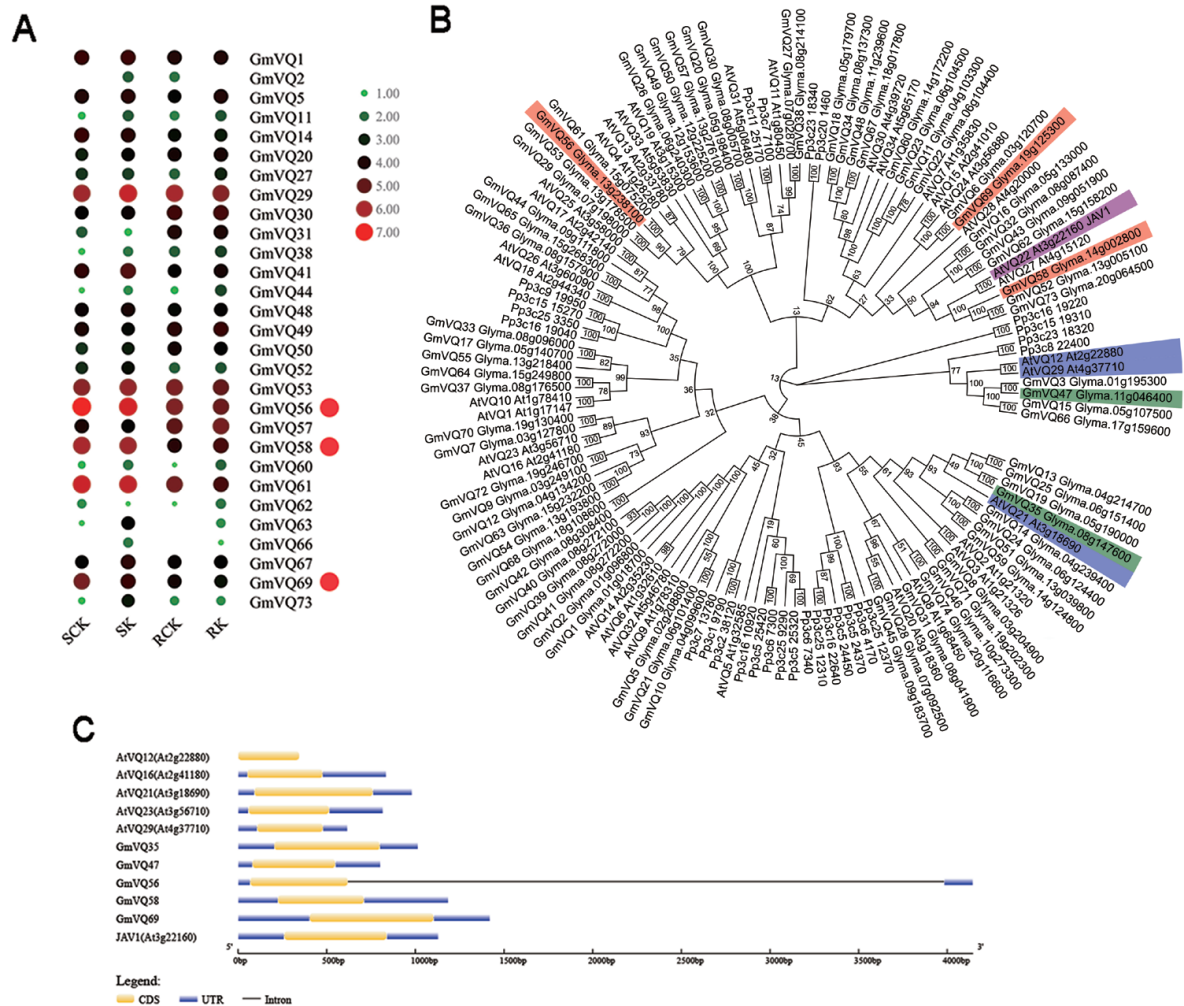
Identification of differentially expressed soybean *VQ* genes. (A) A heatmap was generated based on the gene expression of the genes (listed in Supplementary Table S3). SK, a susceptible soybean line treated by CCW induction; RK, a resistant soybean line treated by CCW induction; SCK and RCK, their corresponding controls; red circles, differentially expressed genes. (B) An NJ phylogenetic tree was constructed using full protein sequences of all *VQ* genes from soybean, Arabidopsis, and *Physcomitrella patens*. Since *GmVQ4* does not exist in the updated soybean reference genome (*Glycine max Wm82.a2.v1*) (Schmutz *et al.*, 2010), its protein sequence was not included in the phylogenetic analysis. Text in red boxes represents proteins encoded by differentially expressed soybean *VQ* genes. Text in green boxes represents disease resistance soybean VQ proteins. Text in blue boxes represents disease resistance Arabidopsis VQ proteins. Text in purple boxes represents the Arabidopsis VQ protein associated with resistance against both insects and pathogens. Bootstrap values from 1000 replicates were used to assess the robustness of the tree. (C) Gene structure analysis of *VQ* genes. CDS, coding sequence; UTR, untranslated region.

Our previous work identified the peak CCW-induced resistance time of the two cultivated soybean lines, and transcriptome analysis was carried out on samples taken during the peak resistance period of the resistant lines (5 d) and the susceptible lines (1 d), with and without CCW induction (Wang *et al.*, 2015*d*). A total of 29 *GmVQ* genes were present in the RNA-seq database (Fig. 1A; Supplementary Table S3). After CCW induction treatment, none of the genes was detected with significant differential expression levels in the resistant lines, and only two genes (*GmVQ63* and *GmVQ73*) were up-regulated in the susceptible lines. However, both of these genes were expressed at a very low level in all treatments (RPKM values ≤15). We then investigated the differentially expressed genes between the non-induced treatments of the resistant and susceptible lines and found that *GmVQ31*, *GmVQ56*, *GmVQ58*, and *GmVQ69* showed differential expression levels. *GmVQ31* transcription was also very low in all treatments (RPKM values ≤15), whereas *GmVQ56*, *GmVQ58*, and *GmVQ69* showed markedly higher expression levels in the susceptible lines than in the resistant lines, and their absolute values of log_2_ ratios were 1.5627, 1.9546, and 1.5662, respectively.

Furthermore, a NJ phylogenetic tree was generated using the protein sequences of all *VQ* genes from soybean, Arabidopsis, and the basal plant *Physcomitrella patens* (Fig. 1B). GmVQ35 and AtVQ21 were clustered together, and GmVQ47 was grouped with AtVQ12 and AtVQ29. GmVQ35, GmVQ47, AtVQ12, AtVQ21, and AtVQ29 are all disease resistance proteins (Petersen *et al.*, 2010; Wang *et al.*, 2015*a*; Zhou *et al.*, 2016). GmVQ58 was grouped with JAV1 (best Arabidopsis hit). *JAV1* functions as a negative regulator of plant defense against both insects and pathogens (Hu *et al.*, 2013*a*). However, the relationship of JAV1 and GmVQ56 with GmVQ69 was distant according to the phylogenetic analysis. Since the *GmVQ58* transcript abundance differed strongly between the resistant and susceptible lines, and its protein was clustered with Arabidopsis AtJAV1, *GmVQ58* was selected for further functional study.

### Sequence analysis of *GmVQ58*

*GmVQ58* is located on chromosome 14 in the soybean genome. The full-length genomic sequence of *GmVQ58* is 1185 bp, including a 486-bp coding region, a 223-bp 5′-untranslated region (UTR), and a 476-bp 3′-UTR. The *GmVQ58* ORF encodes a polypeptide of 161 amino acids with a molecular mass of 17.49 kDa and an isoelectric point of 10.10. Since the majority of *VQ* genes in Arabidopsis, soybean, rice, grape, etc., are intronless (Cheng *et al.*, 2012; Kim *et al.*, 2013; Wang *et al.*, 2015*b*; Zhou *et al.*, 2016), we compared the exon–intron structures of three differentially expressed soybean *VQ* genes (*GmVQ56*, *GmVQ58*, and *GmVQ69*) and eight Arabidopsis or soybean *VQ* genes reported to be associated with biotic stress responses (*AtVQ12*, *AtVQ16*, *AtVQ21*, *JAV1*, *AtVQ23*, *AtVQ29*, *GmVQ35*, and *GmVQ47*) (Petersen *et al.*, 2010; Lai *et al.*, 2011; Hu *et al.*, 2013*a*; Wang *et al.*, 2015*a*; Zhou *et al.*, 2016). With the exception of *GmVQ56*, these 11 genes all had no introns (Fig. 1C).

### *GmVQ58* was highly expressed in leaves and roots and induced by CCW attack

**Fig. 2.**
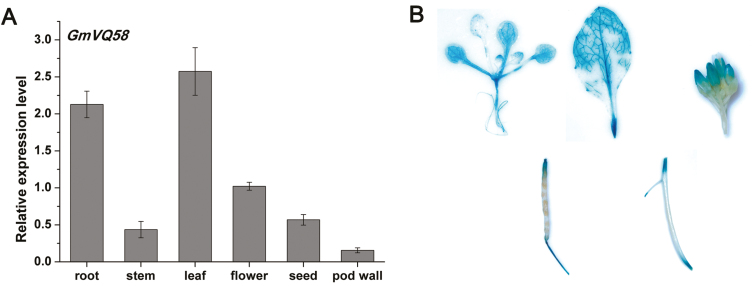
Expression analysis of *GmVQ58* in different soybean tissues. (A) qRT-PCR analysis of *GmVQ58* in leaves, stems, roots, flowers, pod shells, and seeds of soybean (*n*=3). The relative expression levels are normalized to *tubulin* gene and relative to the expression in flowers (relative expression value in flowers=1). Error bars denote ±SE. (B) GUS staining of 7-day-old *GmVQ58*_*pro*_*:GUS* transgenic Arabidopsis seedlings and various tissues from 5-week-old *GmVQ58*_*pro*_*:GUS* Arabidopsis plants.

We investigated the expression levels of *GmVQ58* in different soybean tissues using qRT-PCR. The detection of *GmVQ58* transcripts was highest in leaves and roots (Fig. 2A). To further visualize the expression pattern of *GmVQ58*, we generated transgenic Arabidopsis expressing the GUS reporter gene controlled by the *GmVQ58* promoter. In both 7-day-old seedlings and 5-week-old plants, the GUS reporter gene was highly expressed in leaves and roots, indicating that the soybean *GmVQ58* promoter displays a similar expression pattern in transgenic Arabidopsis (Fig. 2B).

**Fig. 3.**
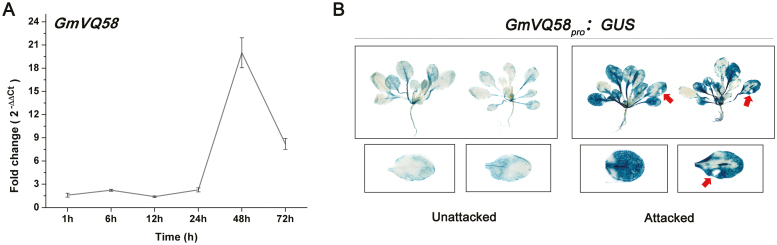
CCW-induced expression of *GmVQ58*. (A) Fold changes of the *GmVQ58* transcript levels at 1, 6, 12, 24, 48, and 72 h after CCW attack compared with normal conditions by qRT-PCR (*n*=3). The relative expression levels are normalized to *tubulin* gene and relative to the expression in control plants at 1 h (relative expression value in control plants at 1 h=1). Error bars denote ±SE. (B) GUS staining of *GmVQ58*_*pro*_*:GUS* transgenic Arabidopsis leaves without and with exposure to CCW attack. Red arrows represent the attacked tissue.

Moreover, we examined *GmVQ58* transcripts at multiple time points after CCW induction by qRT-PCR. As shown in Fig. 3A, the expression of *GmVQ58* was strongly up-regulated after CCW feeding, peaked at 48 h, and then decreased sharply at 72 h in leaves. These results were also demonstrated by GUS staining, as GUS activities were significantly increased in the leaves of 21-day-old *GmVQ58*_*pro*_*:GUS* transgenic Arabidopsis at 1 h after CCW attack (Fig. 3B). Taken together, these results suggested that *GmVQ58* responds to CCW attack and may be involved in plant resistance against CCW.

### GmVQ58 localized in the nucleus

**Fig. 4.**
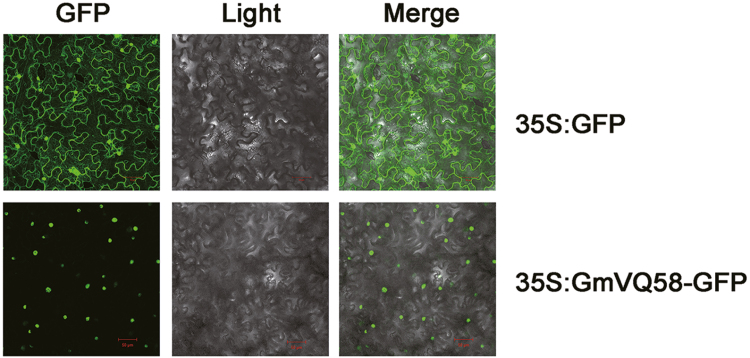
Subcellular localization of GmVQ58 fused to GFP in tobacco mesophyll cells. GFP, GFP fluorescence; Light, bright field; Merge, combination. Scale bars: 50 μm.

To investigate the subcellular localization of GmVQ58 *in vivo*, the CDS of *GmVQ58* fused to GFP was driven by the constitutive CaMV 35S promoter. Then, the construct 35S:GmVQ58-GFP was transiently expressed in the leaves of tobacco (*N. benthamiana*). GFP signals showed that the GmVQ58-GFP fusion protein was only localized in the nuclei of tobacco cells, whereas the empty vector control (35S:GFP) was distributed throughout the cell (Fig. 4).

### *GmVQ58* overexpression suppresses resistance to common cutworm in transgenic Arabidopsis

**Fig. 5.**
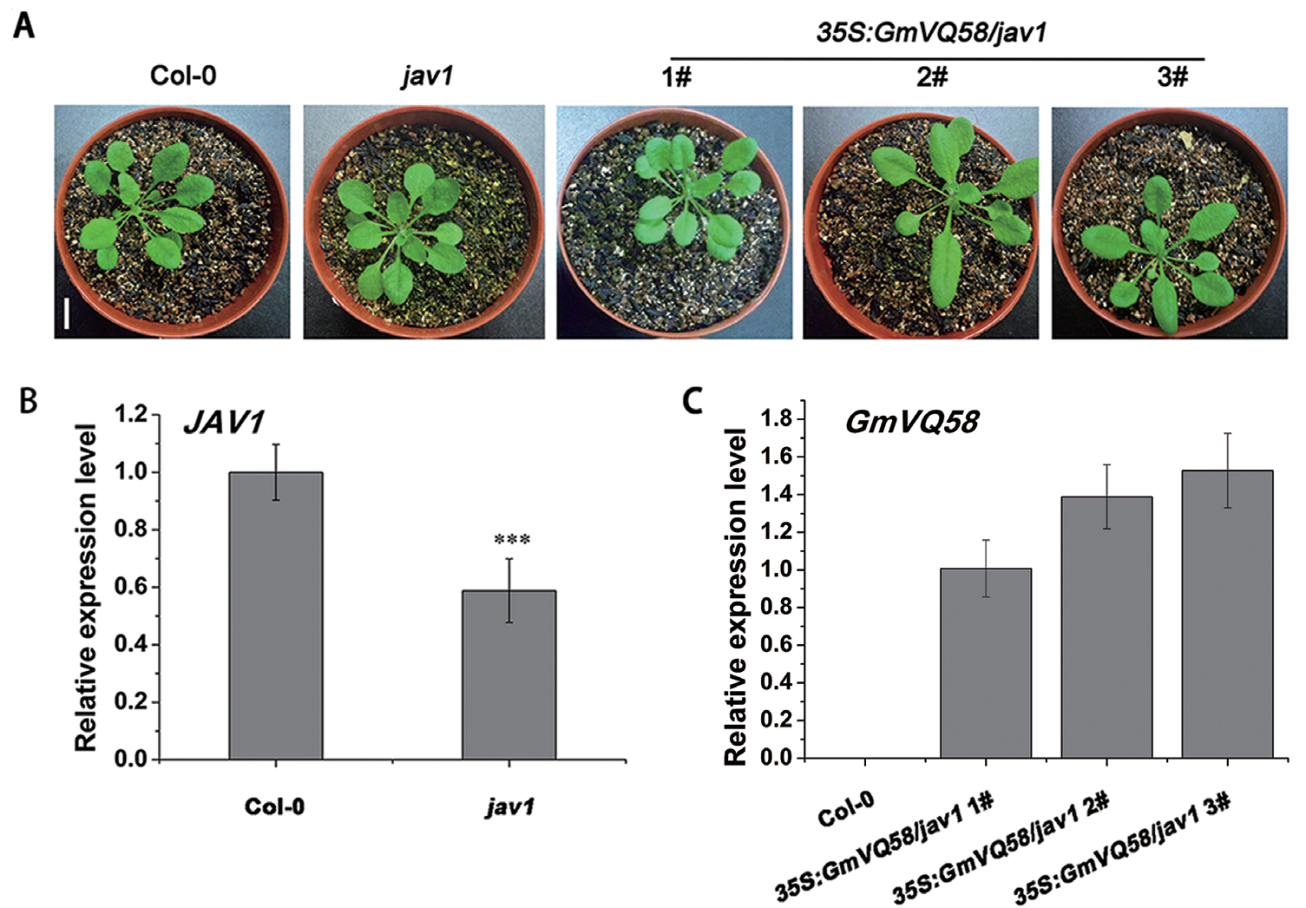
Relative expression levels of *JAV1* and *GmVQ58* in control and transgenic Arabidopsis. (A) Twenty-eight-day-old seedlings of Col-0 plants, the *jav1* mutants, and three *GmVQ58-OE jav1* transgenic lines. Scale bar: 1 cm. (B) Relative expression levels of *JAV1* in Col-0 plants and the *jav1* mutants. The relative expression levels are normalized to *tubulin* gene and relative to the expression in Col-0 (relative expression value in Col-0=1). (C) qRT-PCR analysis of *GmVQ58* in Col-0 plants and three *GmVQ58-OE jav1* transgenic lines. The relative expression levels are normalized to *tubulin* gene and relative to the expression in transgenic Arabidopsis line 1 (*35S:GmVQ58/jav1* 1) (relative expression value in *35S:GmVQ58/jav1* 1#=1). 1#, 2#, and 3# represent three independent *GmVQ58-OE jav1* transgenic lines. A two-tailed *t*-test was used for statistical analysis. *n*=3. ***P<0.001. Error bars denote ±SE.

To identify the role of *GmVQ58* in plant resistance to CCW, we ectopically expressed *GmVQ58* in the *jav1* mutants. *AtJAV1*, the homologous gene of *GmVQ58* in Arabidopsis, functions as a negative regulator in defense against various biotic stresses (Hu *et al.*, 2013*a*). After PCR and qRT-PCR examination, five T_1_ generation *GmVQ58*-OE *jav1* transgenic lines were obtained, and three independent transgenic lines (line 1, line 2, and line 3) with relatively high expression of *GmVQ58* were selected for further phenotypic analysis (see Supplementary Fig. S1). The *jav1* mutants and T_3_ generation *GmVQ58*-OE *jav1* transgenic lines were also examined using PCR (Supplementary Fig. S2) and qRT-PCR. Arabidopsis ecotype Col-0 plants were used as controls. No unique morphological phenotype was observed in the seedlings of Col-0 plants, *jav1* mutants, or three independent *GmVQ58*-OE *jav1* transgenic lines (Fig. 5A), indicating that *GmVQ58* may not play an important role in seedling growth and development. Since the T-DNA was inserted into the *JAV1* gene promoter (Ali *et al.*, 2019), the *jav1* mutant was not a null mutant and displayed a significantly decreased expression of *JAV1* (Fig. 5B). Additionally, *GmVQ58* was expressed in three *GmVQ58-OE jav1* transgenic lines (Fig. 5C).

**Fig. 6.**
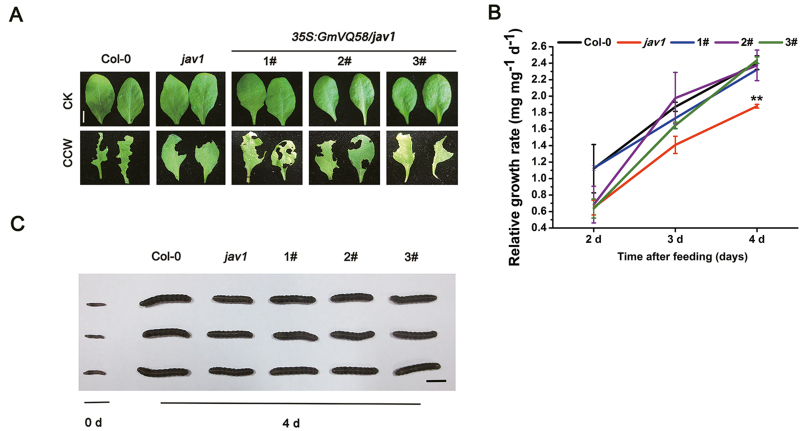
Overexpression of *GmVQ58* in the *jav1* mutants attenuated plant resistance to CCW. (A) Representative rosette leaves of Col-0 plants, the *jav1* mutants and three *GmVQ58-OE jav1* transgenic lines after 24 h of CCW feeding without (CK) or with (CCW) CCW exposure. Scale bar: 4 mm. (B) Relative growth rates of CCW larvae feeding on leaves from Col-0 plants, *jav1* mutants, or three *GmVQ58-OE jav1* transgenic lines after 2, 3, and 4 d of feeding. Two-tailed *t*-tests were used for statistical analysis. *n*=3. **P<0.01. Error bars denote ±SE. (C) Representative CCW larvae taken from rosette leaves of Col-0 plants, the *jav1* mutants, and three *GmVQ58-OE jav1* transgenic lines at days 0 and 4. Scale bar: 1 cm. 1#, 2#, and 3# represent three independent *GmVQ58-OE jav1* transgenic lines.

In the force-feeding trial, 24 Arabidopsis seedlings for each line were used to evaluate insect resistance. The rosette leaves from 28-day-old Col-0 plants, *jav1* mutants, and three *GmVQ58*-OE *jav1* transgenic lines were separately fed to CCW third-instar larvae. After feeding for 4 d, the CCW larvae feeding on the *jav1* mutants devoured a few leaves and grew slowly, whereas Col-0 plants and *GmVQ58*-OE *jav1* transgenic lines promoted larval growth (Fig. 6A, C). Moreover, the RGRs of CCWs feeding on Col-0 plants or the *GmVQ58*-OE *jav1* transgenic lines were significantly higher than those of larvae reared on the *jav1* mutants (Fig. 6B). No significant difference in resistance to CCW was observed between Col-0 plants and the *GmVQ58*-OE *jav1* transgenic lines. Taken together, these results demonstrated that overexpression of *GmVQ58* rescues the susceptibility of the *jav1* mutants to CCW.

### Soybean hairy root transformation confirms the role of *GmVQ58* in resistance against CCW

**Fig. 7.**
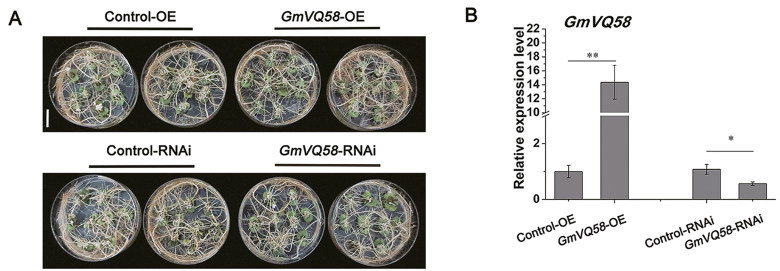
Relative expression levels of *GmVQ58* in transgenic soybean hairy roots. (A) Twenty-five-day-old soybean hairy roots of *GmVQ58*-OE, Control-OE, *GmVQ58*-RNAi, and Control-RNAi genotypes. Scale bar: 2 cm. (B) Relative expression levels of *GmVQ58* in transgenic soybean hairy roots of four genotypes. The relative expression levels are normalized to *tubulin* gene and relative to the expression in Control-OE lines (relative expression value in Control-OE=1). Two-tailed *t*-tests were used for statistical analysis. *n*=3. *P<0.05; **P<0.01. Error bars denote ±SE.

Ectopic expression of *GmVQ58* complemented the *jav1* mutant to restore the Col-0 phenotype. The resistance of complemented Arabidopsis to CCW was similar to that of the Col-0 plants (Fig. 6). Then, we evaluated the function of *GmVQ58* in the soybean system. We generated two constructs, pMDC83-*GmVQ58* and pBI-*GmVQ58*-RNAi, for overexpression (*GmVQ58*-OE) and suppression (*GmVQ58*-RNAi) of *GmVQ58*, respectively. Two constructs with their empty vectors (Control-OE and Control-RNAi) were transformed into soybean hairy roots. In this experiment, we obtained six dishes of transgenic hairy roots for each genotype (Fig. 7A), which were subsequently examined using PCR (Supplementary Fig. S3) and qRT-PCR. Expression analysis showed that the expression level of *GmVQ58* in *GmVQ58*-OE hairy roots was increased by more than 14-fold over that in the Control-OE hairy roots, while the expression level of *GmVQ58* in *GmVQ58*-RNAi hairy roots was 43% lower than that in the Control-RNAi hairy roots (Fig. 7B).

**Fig. 8.**
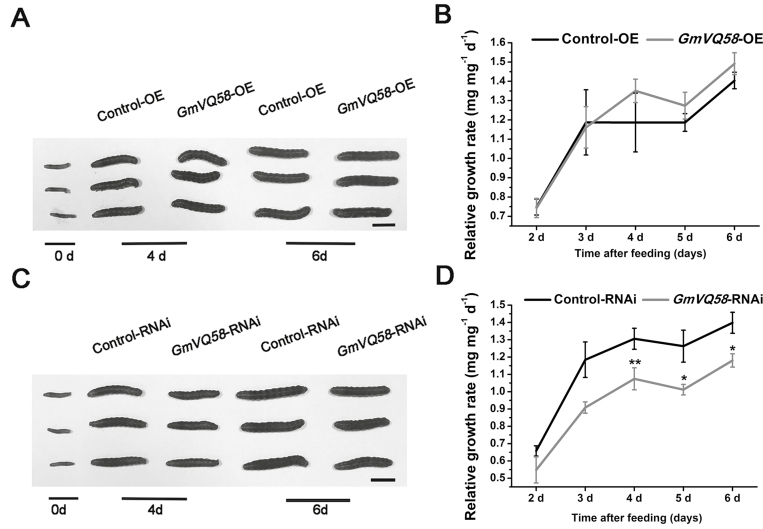
Evaluation of resistance of *GmVQ58* transgenic hairy roots to CCW. (A) Representative CCW larvae taken from *GmVQ58*-OE and Control-OE hairy roots at days 0, 4, and 6. Scale bar: 1 cm. (B) Relative growth rates of CCW larvae feeding on *GmVQ58*-OE or Control-OE hairy roots after 2, 3, 4, 5, and 6 d of feeding. (C) Representative CCW larvae taken from *GmVQ58*-RNAi and Control-RNAi hairy roots at 0, 4, and 6 d. Scale bar: 1 cm. (D) Relative growth rates of CCW larvae feeding on *GmVQ58*-RNAi or Control-RNAi hairy roots after 2, 3, 4, 5, and 6 d of feeding. Two-tailed *t*-tests were used for statistical analysis. *n*=3. *P<0.05; **P<0.01. Error bars denote ±SE.

Furthermore, a 6-d force-feeding trial was performed using transgenic hairy roots. In this experiment, there was no significant difference in resistance to CCW between the *GmVQ58*-OE transgenic hairy roots and the Control-OE roots, although the RGRs of CCWs feeding on the *GmVQ58*-OE transgenic hairy roots were slightly higher than those of CCWs feeding on the Control-OE hairy roots (Fig. 8A, B). However, after feeding for 4 d, the CCW larvae feeding on the *GmVQ58*-RNAi transgenic hairy roots grew more slowly than those feeding on the Control-RNAi hairy roots (Fig. 8C). We compared their RGRs and found that when fed with *GmVQ58*-RNAi hairy roots, the RGRs of CCWs were significantly lower than those of CCWs feeding on the Control-RNAi hairy roots (Fig. 8D). Taken together, these results suggested that silencing *GmVQ58* elevates soybean resistance to CCW.

### GmVQ58 interacts with GmWRKY32

**Fig. 9.**
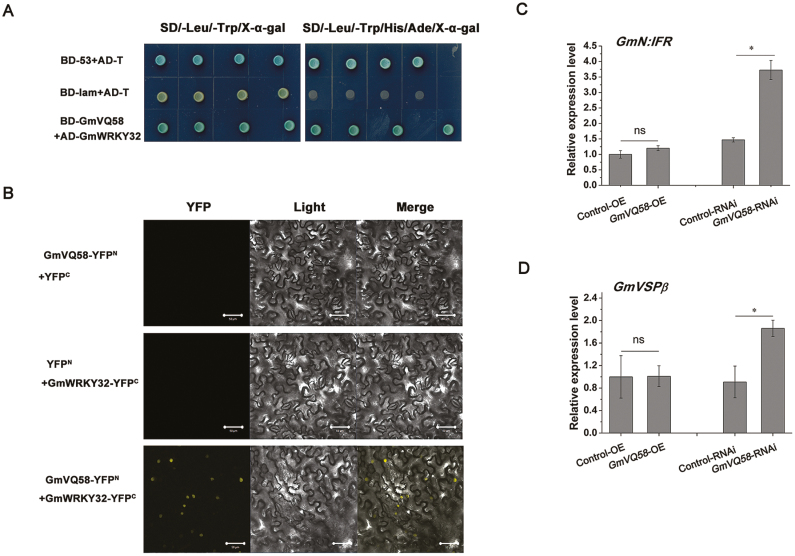
GmVQ58 interacts with GmWRKY32. (A) Y2H assay showed that GmVQ58 interacted with GmWRKY32. BD-53: pGBKT7-53; BD-lam: pGBKT7-lam; BD-GmVQ58: pGBKT7-GmVQ58; AD-T: pGADT7-T; AD-GmWRKY32: pGADT7-GmWRKY32; SD/−Trp/−Leu/X-α-Gal: double-dropout medium supplemented with X-α-Gal; SD/−Trp/−Leu/−His/−Ade/X-α-Gal: quadruple-dropout medium supplemented with X-α-Gal. (B) Bimolecular fluorescence complementation assay showed that GmVQ58 interacted with GmWRKY32. GmVQ58 was fused with YFP^N^ (GmVQ58-YFP^N^), and GmWRKY32 was fused with YFP^C^ (GmWRKY32-YFP^C^). The combinations of GmVQ58-YFP^N^, YFP^C^ and GmWRKY32-YFP^C^, YFP^N^ were used as controls. YFP, YFP fluorescence; Light, bright field; Merge, combination. Scale bars: 50 μm. (C, D) Relative expression levels of *GmN:IFR* (C) and *GmVSPβ* (D) in transgenic soybean hairy roots of four genotypes. The relative expression levels are normalized to *tubulin* gene and relative to the expression in Control-OE lines (relative expression value in Control-OE=1). Two-tailed *t*-tests were used for statistical analysis. *n*=3. ns: not significant; *P<0.05. Error bars denote ±SE.

*GmN*:*IFR* (*Glyma.01g172600*) and *GmVSPβ* (*Glyma.08g200100*), two CCW-responsive genes, positively regulate plant resistance to CCW in tobacco (Wang *et al.*, 2015*d*). Our previous studies showed that GmWRKY32 (Glyma.02g115200) could strongly activate the transcription of *GmN*:*IFR* (8.5-fold) and *GmVSPβ* (4.8-fold) in Arabidopsis protoplasts (Wang *et al.*, 2015*c*). Additionally, several members of the VQ family have been reported to physically interact with WRKY transcription factors to participate in plant defense reactions (Cheng *et al.*, 2012). We then examined the interaction between GmVQ58 and GmWRKY32 using the Y2H system. GmVQ58 was ligated with the GAL4 DNA-binding domain (BD-GmVQ58), and GmWRKY32 was fused to the GAL4 activation domain (AD-GmWRKY32). As shown in Fig. 9A, coexpression of BD-GmVQ58 with AD-GmWRKY32 caused strong activation of α-galactosidase activity, suggesting that GmVQ58 interacts with GmWRKY32 in yeast. The interaction between GmVQ58 and GmWRKY32 in plant cells was subsequently confirmed by bimolecular fluorescence complementation assay. GmVQ58 and GmWRKY32 were fused to the N-terminal and C-terminal fragments of YFP, respectively. YFP fluorescence was observed in the nuclei of tobacco cells co-transformed with the GmVQ58-YFP^N^ and GmWRKY32-YFP^C^ constructs, whereas no YFP fluorescence was detected in the controls (Fig. 9B). All of these results indicated that GmVQ58 physically interacts with GmWRKY32.

Furthermore, we examined the transcript levels of two *GmWRKY32* downstream genes (*GmN*:*IFR* and *GmVSPβ*) in *GmVQ58* transgenic soybean hairy roots by qRT-PCR (Fig. 9C, D). No significant change in their expression was detected between *GmVQ58*-OE roots and Control-OE roots. However, the expression levels of *GmN:IFR* and *GmVSPβ* were higher in the *GmVQ58*-RNAi roots than in the Control-RNAi roots, indicating that the interference of *GmVQ58* increased the expression of *GmN:IFR* and *GmVSPβ* in soybean. Additionally, up-regulated expression of the two genes in *GmVQ58*-RNAi lines suggested that disruption of *GmVQ58* enhances soybean resistance to CCW.

### *GmVQ58* underwent selection during domestication

Insect resistance is a key domestication trait for artificial selection, and related regulatory genes may have been selected during soybean domestication (Zhou *et al.*, 2015). Thus, the expression patterns of *GmVQ58* in our previous RNA-seq data for cultivated soybeans and wild soybeans were compared and are listed in Supplementary Tables S3, S4. Unlike the expression of *GmVQ58* in cultivated soybeans, there was no significant difference in the expression level of its homologous gene *Glysoja.14g037272* between the resistant and susceptible wild soybean lines under non-induced treatments, although *Glysoja.14g037272* was up-regulated at 3 d after CCW attack in resistant wild soybean lines (Du *et al.*, 2019).

Variation in promoter regions may affect gene expression patterns. GUS staining showed that the 2184-bp region upstream of the ATG of *GmVQ58* had promoter activity (Figs 2B, 3B). Eight SNPs were detected in the 2184-bp upstream region of *GmVQ58* in 302 soybean accessions, including 62 wild soybeans, 130 landraces, and 110 cultivars (Supplementary Fig. S4), and the relative conservation value of the *GmVQ58* promoter sequences among accessions was 94% (Supplementary Table S5; Zhou *et al.*, 2015). By detecting the sequence diversity within the *GmVQ58* promoter between accessions in each subpopulation, we found that the nucleotide variation (π) in wild soybeans was 0.000967, whereas the π values of the landraces (0.000591) and cultivars (0.000608) were relatively low (Table 1), suggesting that wild soybeans had higher promoter diversity than did the others. Moreover, Tajima’s *D* values of the wild soybeans, landraces, and cultivars were 1.594, 0.367, and 0.002, respectively, implying that there were more rare alleles in the cultivated soybeans than in the wild ones. Taken together, these results suggested that the *GmVQ58* promoter might have been selected during soybean domestication.

**Table 1.** Summary of the sequence diversity of *GmVQ58* promoter in wild, landrace, and cultivated soybeans

Sequence locus	Wild		Landrace		Cultivar	
	π	Tajima’s *D*	π	Tajima’s *D*	π	Tajima’s *D*
Promoter	0.000967	1.594	0.000591	0.367	0.000608	0.002

## Discussion

The discovery of plant endogenous insect resistance genes is undeniably of great significance for understanding the co-evolution of plants and pests and improving crop resistance to insects. Transcriptome sequencing has been successfully used for the identification of new plant resistance genes (Huang *et al.*, 2015; Hickman *et al.*, 2017). In this study, a new VQ motif-containing gene, *GmVQ58*, was analysed via various molecular and genetic approaches.

### *GmVQ58* is highly responsive to CCW attack, and silencing the gene elevates soybean defense against CCW

*VQ* genes play critical roles in plant defense against biotic stresses. Here, we analysed a soybean *VQ* gene, *GmVQ58*. The gene was highly responsive to CCW attack (Fig. 3). Constitutive expression of the gene rescued the susceptibility of the *jav1* mutants to CCW in Arabidopsis (Fig. 6). Overexpression of the gene did not significantly affect soybean resistance to CCW, whereas resistance was significantly enhanced in *GmVQ58*-RNAi lines (Fig. 8). Taken together, these results suggested that *GmVQ58* plays a role in plant defense against CCW.

*VQ* genes belong to a polygenic family. Most *VQ* genes participate in plant pathogen resistance, such as Arabidopsis *AtVQ12*, *AtVQ16*, *AtVQ21*, *AtVQ23*, and *AtVQ29* (Petersen *et al.*, 2010; Lai *et al.*, 2011; Wang *et al.*, 2015*a*) and 13 rice *VQ* genes (Li *et al.*, 2014). Seventy-four genes of the soybean *VQ* gene family have been predicted (Wang *et al.*, 2014). Similar to their homologs in other plants, *GmVQ35* and *GmVQ47* regulate plant resistance against *B*. *cinerea* in transgenic Arabidopsis (Zhou *et al.*, 2016). In this study, the two genes were not present in soybean transcriptomic data (Fig. 1A; Supplementary Table S3), and their proteins were respectively grouped with different Arabidopsis VQ proteins related to resistance against pathogens (Fig. 1B). However, GmVQ58 as well as GmVQ52 and GmVQ73 was classified with AtVQ22 (JAV1) (Fig. 1B). *GmVQ52* did not show differential transcript abundance between resistant and susceptible soybean lines, and *GmVQ73* displayed low expression in all treatments (Fig. 1A; Supplementary Table S3). These results also indicated that the function of *GmVQ58* might be different from that of its homologs in soybean. Furthermore, in addition to playing a role in plant resistance against several insects, *JAV1* negatively regulates plant resistance against pathogens (Hu *et al.*, 2013*a*). Whether *GmVQ58* has the same function as *JAV1* needs to be studied in the future.

### Interference of *GmVQ58* enhances the expression of two resistance genes downstream of GmWRKY32

To date, several members of the VQ family have been reported to physically interact with a number of proteins, especially WRKYs. Many studies have shown that WRKYs play multiple roles in responses to biotic and abiotic stresses. In Arabidopsis, AtVQ23 and AtVQ16 function as regulators of the transcription factor AtWRKY33 and positively control plant resistance against *B*. *cinerea* (Lai *et al.*, 2011). AtVQ21 was also found to interact with AtWRKY33 and may affect WRKY-regulated resistance gene expression (Andreasson *et al.*, 2005). In banana fruit, MaVQ5 physically interacted with MaWRKY26 to regulate its transactivation of jasmonic acid biosynthetic genes (Ye *et al.*, 2016).

In our previous studies, *GmN:IFR* and *GmVSPβ* positively regulated plant defense against CCW in tobacco, and GmWRKY32 could strongly activate the transcription of *GmN:IFR* and *GmVSPβ* in Arabidopsis protoplasts (Wang *et al.*, 2015*c*,*d*). A total of 176 *WRKY* genes have been identified in the soybean genome and can be divided into three groups, namely groups I, II, and III. The group II genes are further divided into five subgroups, namely IIa, IIb, IIc, IId, and IIe (Song *et al.*, 2016). *GmWRKY32* is a member of soybean *WRKY* group IIc, and its Arabidopsis ortholog, *AtWRKY57* (*At1g69310*), has been implicated in plant biotic stress (*B*. *cinerea* resistance) and abiotic stress (drought tolerance) (Jiang *et al.*, 2016; Jiang and Yu, 2016). In this study, we showed that GmVQ58 physically interacted with GmWRKY32, and the expression of *GmN:IFR* and *GmVSPβ* was up-regulated in *GmVQ58*-RNAi lines (Fig. 9). Based on these results, we hypothesize that the interaction between GmVQ58 and GmWRKY32 might repress the transcriptional activation activity of GmWRKY32 on *GmN:IFR* and *GmVSPβ*. In addition, the *GmVQ58* gene might function in soybean insect resistance in other ways. To better understand the insect resistance mechanism of the *GmVQ58* gene in soybean, more studies need to be considered in the future, such as the construction of *GmVQ58* overexpression and silent soybean lines.

### The promoter diversity of *GmVQ58* is relatively high in wild soybeans

We cloned the 2184-bp region upstream of the ATG of *GmVQ58*. The *GmVQ58* promoter could activate the expression of the GUS gene in multiple tissues in transgenic Arabidopsis (Fig. 2B). After CCW attack, the GUS gene controlled by the *GmVQ58* promoter was up-regulated in transgenic Arabidopsis (Fig. 3B). These results indicated that the *GmVQ58* promoter has transcriptional activity. In addition, the expression patterns of *GmVQ58* in cultivated soybeans and wild soybeans were different (Supplementary Tables S3, S4). Therefore, sequence diversity in the promoter of *GmVQ58* was analysed in 302 soybean accessions (Table 1). In the population, wild soybeans had the highest variation, followed by landraces and improved cultivars, suggesting that the promoter region of *GmVQ58* might have been selected during soybean domestication. Domestication is a complex process that includes both natural and artificial selection. The diversity of half of the resistance-related genes decreased in this process (Zhao *et al.*, 2015). It is unknown whether beneficial *GmVQ58* alleles were retained or lost in cultivated soybeans, and additional work is needed, such as the evaluation on CCW resistance of the 302 soybean accessions.

## Supplementary data

Supplementary data are available at *JXB* online.

Fig. S1. Identification of T_1_ generation transgenic Arabidopsis plants by PCR and qRT-PCR.

Fig. S2. Identification of T_3_ generation transgenic Arabidopsis plants by PCR.

Fig. S3. Identification of transgenic soybean hairy roots by PCR.

Fig. S4. Polymorphisms detected within the promoter region of *GmVQ58* in 302 soybean accessions.

Table S1. Primer pairs used in soybean.

Table S2. Primer pairs used in Arabidopsis.

Table S3. List of all *VQ* genes present in the RNA-seq database for cultivated soybeans and their expression patterns.

Table S4. List of all *VQ* genes present in the RNA-seq database for wild soybeans and their expression patterns.

Table S5. Sequence alignment results of the promoter region of *GmVQ58* in 302 soybean accessions.

eraa095_suppl_Supplementary_Figures_S1_S4

eraa095_suppl_Supplementary_Tables_S1_S5

## Author contributions

HW and DY designed the research. XL and DH conducted the experiments. RQ, QD, and LC assisted with performing the experiments. HD, HY, JW, and FH analysed the data. XL wrote the manuscript. HW and DY revised the manuscript. All authors read and approved the final manuscript.

## Abbreviations

CCW: common cutworm
CDS: coding sequence
GFP: green fluorescent protein
GUS: β-glucuronidase
NJ: neighbor-joining
qRT-PCR: quantitative real-time polymerase chain reaction
RGR: relative growth rate
RPKM: reads per kilobase per million mapped reads
SNP: single-nucleotide polymorphism
UTR: untranslated region
Y2H: yeast two-hybrid
YFP: yellow fluorescent protein
