# Supplementary material for: Knockdown of GmVQ58 encoding a VQ motif-containing protein enhances soybean resistance to the common cutworm (Spodoptera litura Fabricius)
Source: J Exp Bot. 2020 Feb 20;71(10):3198–210. doi: 10.1093/jxb/eraa095 (PMC7475176; doi:10.1093/jxb/eraa095)
Supplement: eraa095_suppl_Supplementary_Figures_S1_S4 [file eraa095_suppl_supplementary_figures_s1_s4.pdf]

Knock down of *GmVQ58* encoding a VQ motif-containing protein enhances soybean resistance to the common cutworm (*Spodoptera litura* Fabricius)

Running title: *GmVQ58* is involved in soybean insect resistance

Authors:

Xiao Li[1], Rui Qin[1], Qing Du[1], Linyan Cai[1], Dezhou Hu[1], Haiping Du[2], Hui Yang[2], Jiao Wang[1], Fang Huang[1], Hui Wang[1]* and Deyue Yu[1,2]*

Institution address:

[1] National Center for Soybean Improvement, National Key Laboratory of Crop Genetics and Germplasm Enhancement, Jiangsu Collaborative Innovation Center for Modern Crop Production, Nanjing Agricultural University, Nanjing 210095, China

[2] School of Life Sciences, Guangzhou University, Guangzhou 510006, China

* Corresponding authors:

Hui Wang, Tel: +86-25-84399527; Fax: +86-25-84396410; e-mail: wanghui0@njau.edu.cn.

Deyue Yu, Tel: +86-25-84396410; Fax: +86-25-84396410; e-mail: dyyu@njau.edu.cn.

Fig. S1

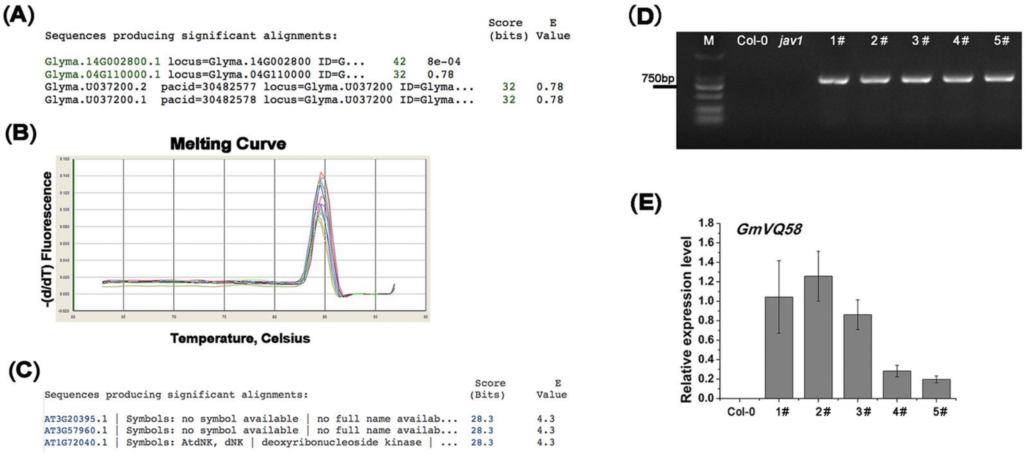

Supplementary Fig. S1. Identification of T_1 generation transgenic *Arabidopsis* plants by PCR and qRT-PCR. (A) BLAST analysis against soybean transcript sequences using qRT-PCR primers for the *GmVQ58* gene. (B) Melting curve analysis of the qRT-PCR primers for the *GmVQ58* gene in soybean. (C) BLAST analysis against *Arabidopsis* transcript sequences using qRT-PCR primers for the *GmVQ58* gene. (D) Identification of T_1 generation *GmVQ58* transgenic *Arabidopsis* plants via amplification of an 855-bp fragment of genomic DNA. (E) qRT-PCR analysis of *GmVQ58* expression in Col-0 plants and T_1 generation *GmVQ58* transgenic lines. The relative expression levels are normalized to *tubulin* gene and relative to the expression in transgenic *Arabidopsis* line 1 (*35S:GmVQ58/jav1* 1#) (relative expression value in *35S:GmVQ58/jav1* 1# = 1). M: DNA Marker DL 2000; Col-0: *Arabidopsis* ecotype Col-0; *jav1*: the *jav1* mutants; numerical code: different T_1 generation *GmVQ58*-OE *jav1* transgenic lines. N = 3. Error bars denote ±SE.

Fig. S2

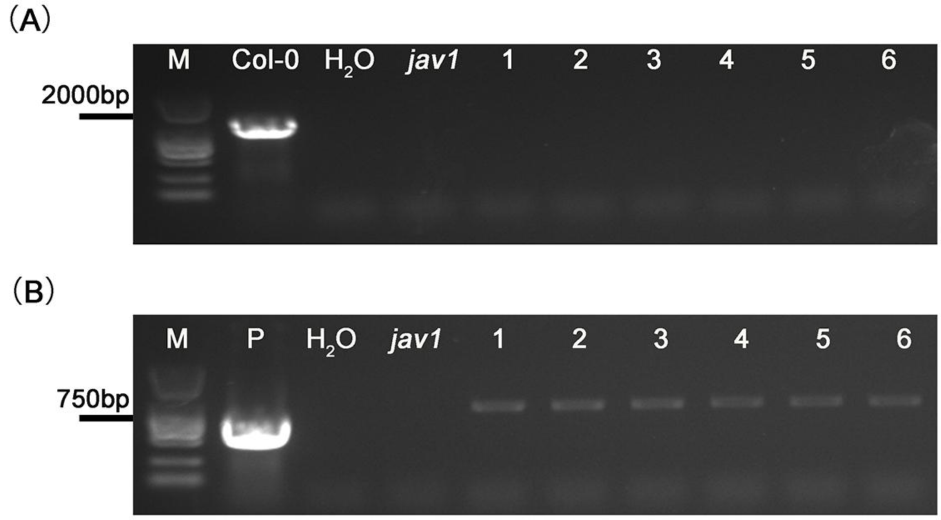

Supplementary Fig. S2. Identification of T_3 generation transgenic *Arabidopsis* plants by PCR. (A) Identification of the *jav1* mutants by PCR. Primers were designed according to sequences near the T-DNA insertion site. Within the specified extension time, the 1329-bp fragment could not be amplified in mutant plants carrying a T-DNA insertion in the *JAV1* promoter, whereas 1329-bp fragment could be amplified in Col-0 plants. (B) Identification of T_3 generation *GmVQ58* transgenic *Arabidopsis* plants by amplifying an 855-bp fragment of genomic DNA. M: DNA Marker DL 2000; Col-0: *Arabidopsis* ecotype Col-0; *jav1*: the *jav1* mutants; P: positive control (pMDC83-*GmVQ58* plasmid DNA); numerical code: different T_3 generation *GmVQ58*-OE *jav1* transgenic plants; H_2O: blank control.

Fig. S3

(A)

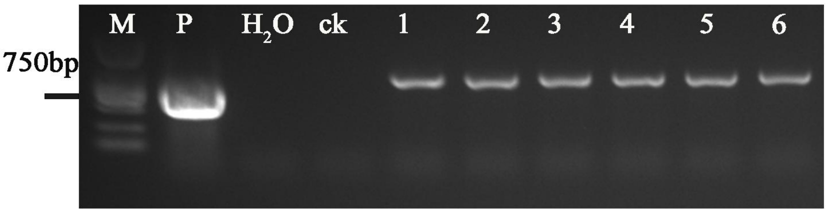

(B)

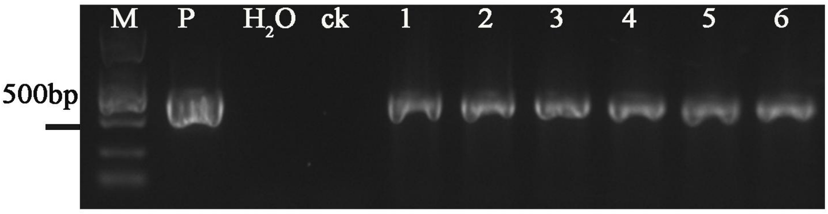

Supplementary Fig. S3. Identification of transgenic soybean hairy roots by PCR. (A) PCR amplification of *GmVQ58*-OE transgenic hairy roots and Control-OE hairy roots was performed to detect an 855-bp fragment. M: DNA Marker DL 2000; P: positive control (pMDC83-*GmVQ58* plasmid DNA); H_2O: blank control; ck: soybean hairy roots transformed with the control vector pMDC83; numerical code: different lines transformed with the pMDC83-*GmVQ58* plasmid. (B) PCR amplification of *GmVQ58*-RNAi transgenic hairy roots and Control-RNAi hairy roots was performed to detect a 625-bp fragment. M: DNA Marker DL 2000; P: positive control (pB7GWIWG2(II)-*GmVQ58* plasmid DNA); H_2O: blank control; ck: soybean hairy roots transformed with the control vector pB7GWIWG2(II); numerical code: different lines transformed with the pB7GWIWG2(II)-*GmVQ58* plasmid.

Fig. S4

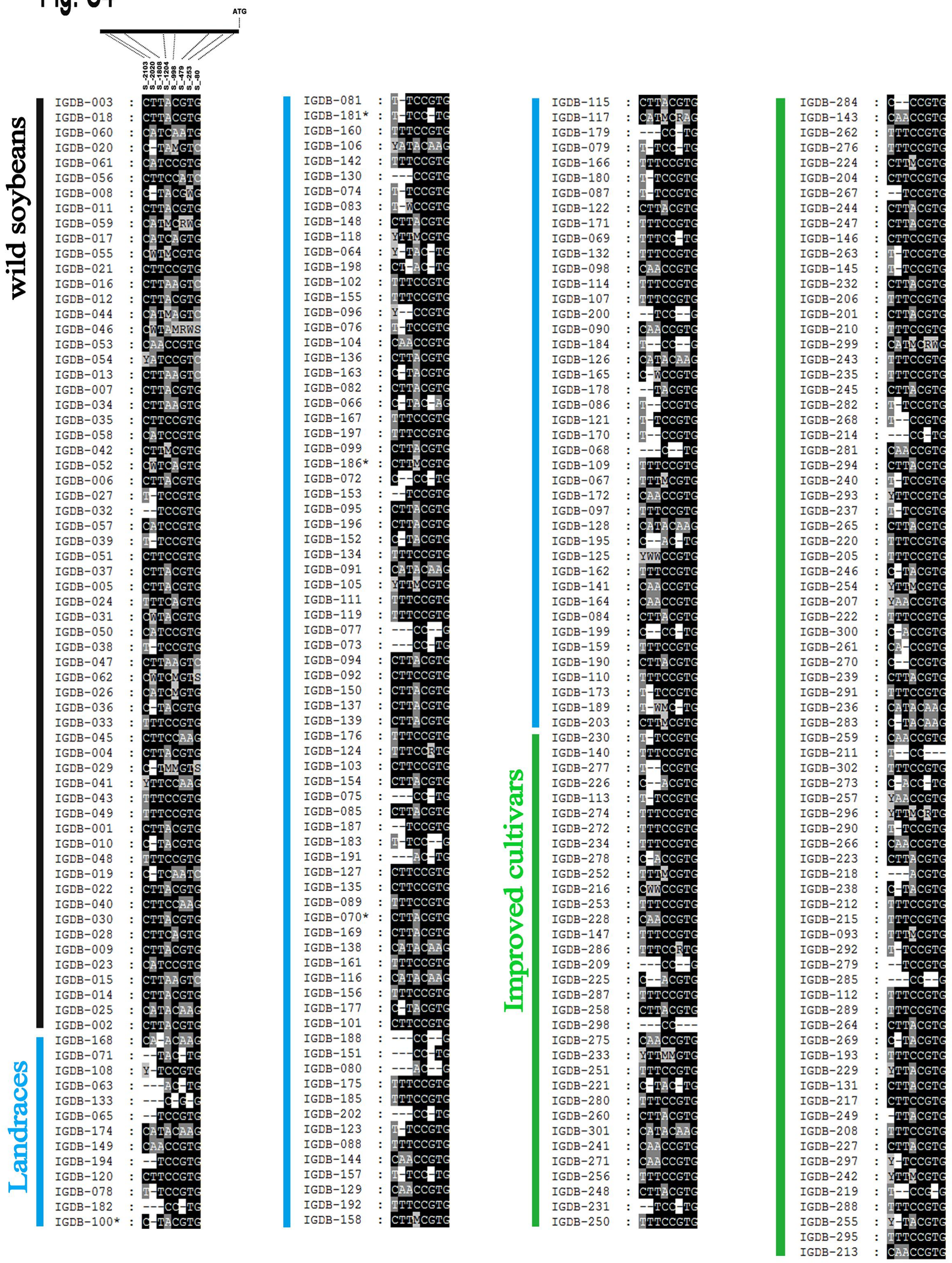

Supplementary Fig. S4. Polymorphisms detected within the promoter region of *GmVQ58* in 302 soybean accessions. The 302 soybean accessions include 62 wild soybeans, 130 landraces and 110 improved cultivars. Eight SNPs (Table S5), S_-2103 (located 2103 bp upstream of the ATG of *GmVQ58*), S_-2020, S_-1808, S_-1204, S_-998, S_-479, S_-253 and S_-80, were

identified on the basis of the previously published whole-genome resequencing data (Zhou *et al*., 2015). The physical position of each SNP is shown above the plot. The degenerate bases R, Y, M, S and W represent A/G, C/T, A/C, G/C and A/T, respectively. "-" represents a deletion mutation.
